# Morphological and molecular characteristics of *HER2* amplified urothelial bladder cancer

**DOI:** 10.1007/s00428-015-1729-4

**Published:** 2015-03-26

**Authors:** J. Tschui, E. Vassella, N. Bandi, U. Baumgartner, V. Genitsch, D. Rotzer, R. Seiler, G. N. Thalmann, A. Fleischmann

**Affiliations:** 1 Institute of Pathology, University of Bern, Bern, Switzerland; 2 Department of Urology, University Hospital of Bern, Bern, Switzerland; 3 Institute of Pathology, University of Bern, Murtenstr.31, 3010 Bern, Switzerland; 4 Institute of Pathology, Postfach 100, 8596 Münsterlingen, Switzerland

**Keywords:** HER2, Amplification, Bladder cancer, Histopathology

## Abstract

Several (pre-) clinical trials are currently investigating the benefit of HER2-targeted therapy in urothelial bladder cancer (UBC). Patients with HER2 amplified UBC could potentially profit from these therapies. However, little is known about histomorphology, HER2 protein expression patterns and occurrence of alterations in the *HER2* gene in their tumors. Among 150 metastasizing primary UBC, 13 *HER2* amplified tumors were identified. Their histopathological features were compared with 13 matched, non-amplified UBC. HER2 protein expression was determined by immunohistochemistry. The 26 tumors were screened for mutations in exons 19 and 20 of the *HER2* gene. UBC with *HER2* amplification presented with a broad variety of histological variants (median 2 vs. 1), frequently featured micropapillary tumor components (77 % vs. 8 %) and demonstrated a high amount of tumor associated inflammation. Immunohistochemically, 10 of 13 (77 %) *HER2* amplified tumors were strongly HER2 protein positive. Three tumors (23 %) were scored as HER2 negative. One of the *HER2* amplified tumors harbored a D769N mutation in exon 19 of the *HER2* gene; all other tested tumors were wild type. In conclusion, *HER2* amplified UBC feature specific morphological characteristics. They frequently express the HER2 protein diffusely and are, therefore, promising candidates for HER2 targeted therapies. The detection of mutations at the HER2 locus might add new aspects to molecular testing of UBC.

## Introduction

The human epidermal growth factor receptor 2 (HER2/neu, erbB2) constitutes, together with HER1 (EGFR, erbB1), HER3 (erbB3), and HER4 (erbB4), the type I group of 20 families of receptor tyrosine kinases. HER2 is a transmembrane 185 kDa protein; its encoding gene is on chromosome 17q21 [1]. The orphan HER2 without known ligand acts as co-receptor for heterodimer formations with the other EGFR family members [2]. These receptor heterodimers are drivers of cellular proliferation [3], inhibit apoptosis [4], and promote angiogenesis [5].

HER2 overexpression characterizes particularly aggressive cancer types of various origin that share poor outcome [6]. Originally detected in a subset of breast cancer [7], amplification of the HER2 gene is the primary mechanism for protein overexpression [8]. At present, targeted anti-HER2 therapies are established clinical routine for HER2 overexpressing/amplified carcinomas of the breast [9] and stomach [10]. Recent works have evaluated HER2 status in urothelial bladder cancer (UBC) in order to assess the therapeutic potential of this target, demonstrating significant protein overexpression (score 2+ or 3+) or gene amplification in approximately 10 % of the tumors [11–15]. In addition, several phase II and even phase III trials are currently investigating the possible benefit of HER2 targeted therapies for patients with UBC (http://clinicaltrials.gov: NCT00151034, NCT00004856, NCT00005831. https://www.clinicaltrialsregister.eu: EudraCT: 2007-001826-28).

Considering these developments, pathologists will presumably have to identify UBC with *HER2* amplification for personalized treatment in the nearer future. As only little is known about the histomorphology of these cases, better knowledge hereof might facilitate their identification. Morphological preselection before accomplishing additional examinations by immunohistochemistry or molecular procedures may even be interesting from an economical point of view. Finally, HER2 expression of amplified UBC has not been described in detail and the presence of additional mutations is largely unknown. We evaluated these open questions in a high-risk cohort of advanced metastasizing UBC.

## Methods

### Patients

Our cohort comprised of 150 bladder cancer patients (29 females and 121 males) treated by standardized, extended bilateral pelvic lymphadenectomy with cystectomy as a single procedure at the Department of Urology, University Hospital of Bern, Switzerland. No neoadjuvant therapy was given. Median age at surgery was 67 years (range 35–89); most primary tumors were advanced (pT1, *n* = 4; pT2, *n* = 17; pT3, *n* = 92; and pT4, *n* = 37).

### Pathological techniques

The opened bladder specimens were fixed overnight in neutral buffered formalin and processed at the Institute of Pathology, University of Bern. The tumor samples tested for molecular alterations were collected in accordance with the required international ethical guidelines including approval by the Institutional Review Board at the Institute of Pathology, University of Bern.

### Identification of HER2 amplified and non-amplified cancers

A TMA with the 150 UBC was constructed and evaluated for *HER2* amplification by fluorescence in situ hybridization. These results were published previously [12]. In the current study, the 13 amplified tumors (HER2/CEP17 ratio ≥ 2.2 [16]) were further investigated and their histopathological characteristics compared with 13 non-amplified UBC (HER2/CEP17 ratio < 1.8; [16]) from our cohort, matched by age, pT stage, and operation date.

### Morphological evaluation

All *HER2* amplified and non-amplified urothelial carcinomas were evaluated by two independent investigators (JT and AF) for numbers and percentage of histomorphological variants [17] present in the tumors and the final result was formulated as a consensus. These numbers and percentages in each subgroup were added up for comparison. The amount of tumor-associated inflammation was graded from a score zero to three (negative, mild, moderate, and strong) for each case based on the spectrum observed over all tumors.

### Immunohistochemistry and in situ hybridization

Immunohistochemistry for the detection of HER2 expression was performed on one representative cross sectional slide per tumor, displaying a maximum of tumor tissue mass. The original HercepTest (DAKO, Glostrup Denmark) was used for immunohistochemical stains which were performed according to the manufacturer’s protocol. HER2 protein expression per cross sectional slide was classified according to the modified DAKO criteria [16]: negative (0/1+), weakly positive (2+), and positive (3+) with a cut-off for score 3+ for a more than 30 % strong complete membranous staining of the tumor cells.

For comparison of Her2 protein phenotype and amplification status, dual in situ hybridization (ISH) was performed on one large section. The *HER2* gene is detected by a dinitrophenyl (DNP) labeled probe and visualized utilizing VENTANA *ultra*View Silver ISH DNP (black signals) Detection. The chromosome 17 centromere is targeted with a digoxigenin (DIG) labeled probe and detected using VENTANA *ultra*View Red ISH DIG detection (red signals). Detailed instructions for hybridization procedures are provided by the manufacturer.

### *HER2* mutation analysis

Genomic DNA was obtained from all amplified tumor tissues by overnight digestion with proteinase K at 55 °C followed by DNA extraction using the BioRobot EZ1 workstation (Qiagen). Intron-based primers were used to amplify exon 19 and 20 encompassing the main hot spot of activating mutations of the *HER2* gene [18, 19]. The primer sequences were as follows: forward primer exon 19, 5′-CCCACGCTCTTCTCACTCAT-3′; reverse primer exon 19; 5′-TCCTTCCTGTCCTCCTAGCA-3′; forward primer exon 20, 5′-TGGTCTCCCATACCCTCTCA-3′; and reverse primer exon 20, 5′-CAAAGAGCCCAGGTGCATA-3′. Sequence analysis was performed using the 3500 genetic analyzer (Applied Biosystems). In addition, exon 20 was analyzed for in-frame-insertions by capillary electrophoresis using a 6-carboxy-fluorescine labeled forward primer and the same reverse primer.

## Results

### Morphological features of HER2 amplified and non-amplified urothelial bladder cancer

**Fig. 1:**
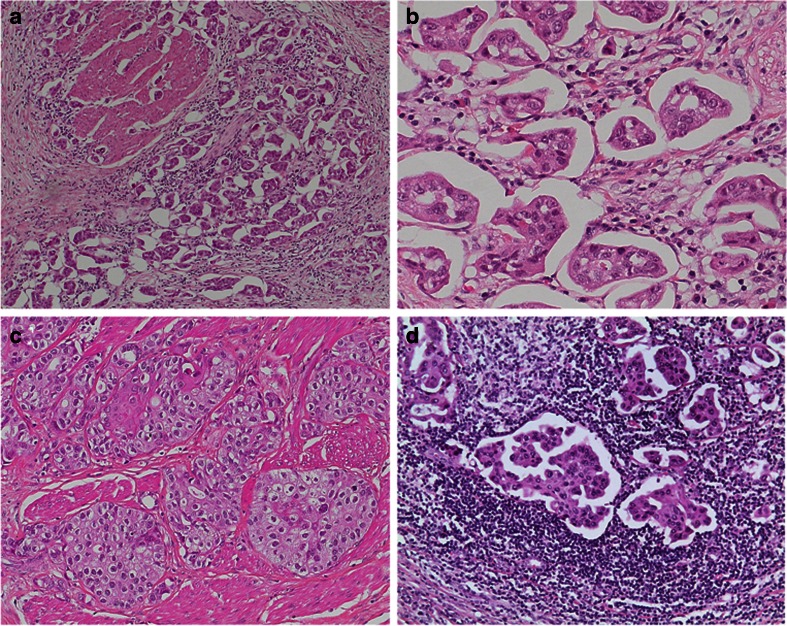
*HER2* amplified urothelial bladder cancers often show micropapillary morphology (**a**, **b** HE × 10 and × 40) throughout the tumor or together with other components like the nested variant (**c** HE × 20). *HER2* amplified urothelial bladder cancer often presents with marked tumor-associated chronic inflammation (**d** HE × 20)

A hallmark of the *HER2* amplified tumor group was micropapillary growth, which was present in 10 out of 13 cases (77 %): three tumors (23 %) were purely (100 %), one tumor (8 %) extensively (80 %), and six tumors (46 %) focally (up to 10 %) micropapillary. When related to the entire tumor mass within the *HER2* amplified group, the micropapillary proportion accounted for 32.7 % of this tumor mass (Figs. 1a, b and 3). *HER2* amplified tumors often presented with components of UBC variants (median 2 per tumor) including the nested variant (Fig. 1c), two tumors (15 %) were composed of three different components. Finally, tumor-associated inflammation was high (mean score 1.9) and showed both intratumoral and peritumoral infiltrates (Fig. 1d).

**Fig. 2:**
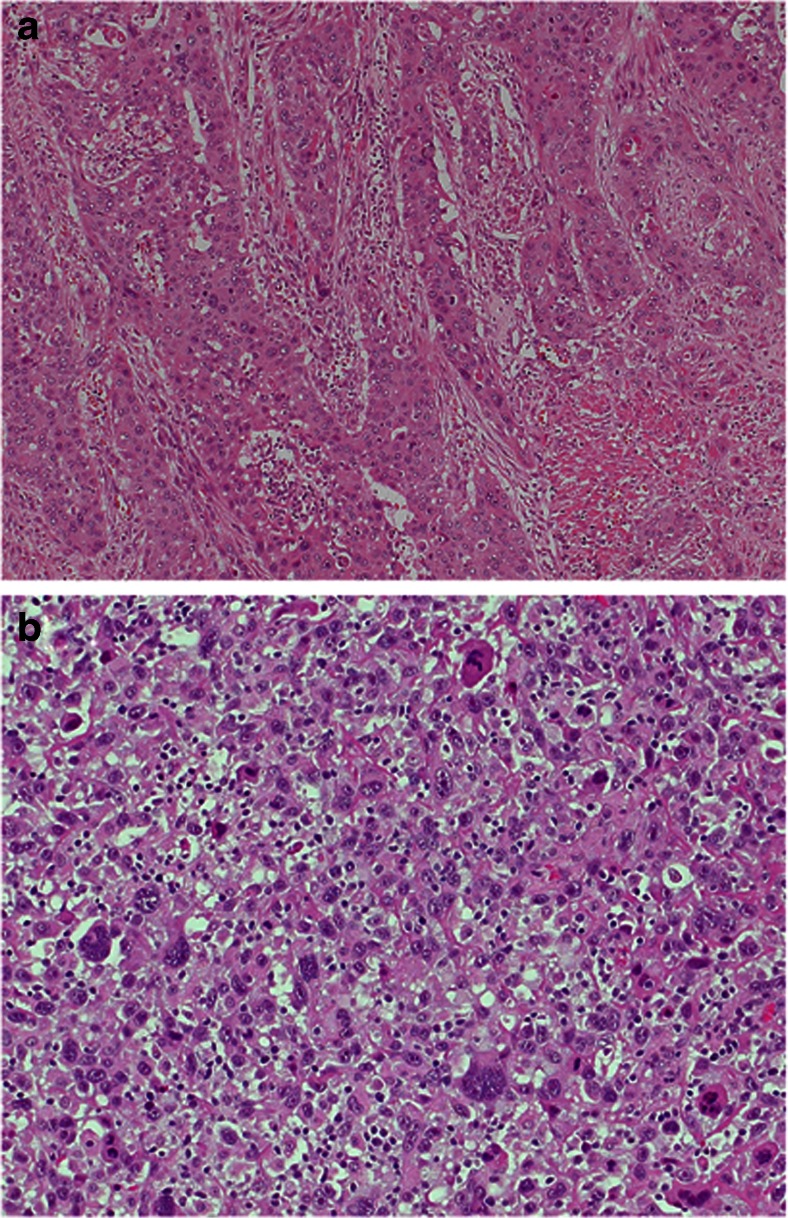
*HER2* non-amplified urothelial bladder cancers mostly show conventional solid morphology (**a** HE × 10); **b** sarcomatoid variant (HE × 20)

**Fig. 3:**
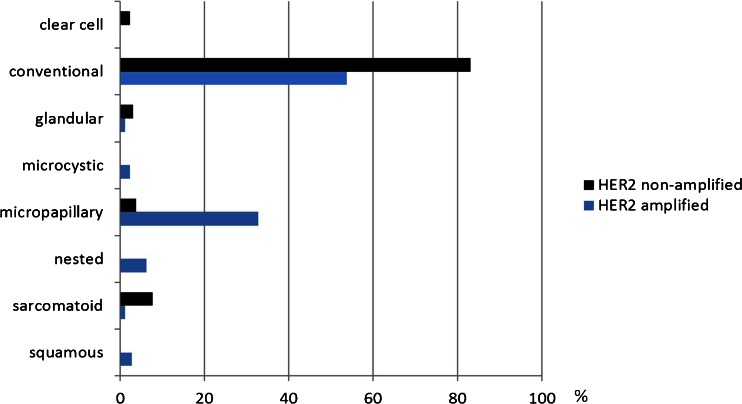
Comparison of *HER2* amplified and non-amplified urothelial bladder cancers (*n*=13 each) in regard to their morphology (the tumor components are given as percentage in relation to the total tumor mass in each group): micropapillary architecture is a key feature of *HER2* amplified urothelial bladder cancer

In contrast, the *HER2* non-amplified group showed mostly conventional UBC: nine tumors (69 %) were purely (100 %) and three tumors (23 %) were extensively (60–70 %) of this type (Fig. 2a). One tumor (8 %) featured a purely sarcomatoid growth pattern (100 %, Fig. 2b) with accompanying urothelial carcinoma in situ and one (8 %) had micropapillary components (40 % of the otherwise conventional UBC), which accounted for 3 % of the total tumor mass in this group. Moreover, tumor-associated inflammation (mean score 1.2) was scarce compared to the amplified group. The trend for more monophasic growth in *HER2* non-amplified compared to *HER2* amplified tumors was reflected in a lower median number of morphologically different tumor components per tumor (1 vs. 2 in the *HER2* amplified group) and the absence of tumors with more than two components.

### Immunohistochemistry and ISH of HER2 amplified UBC

**Fig. 4:**
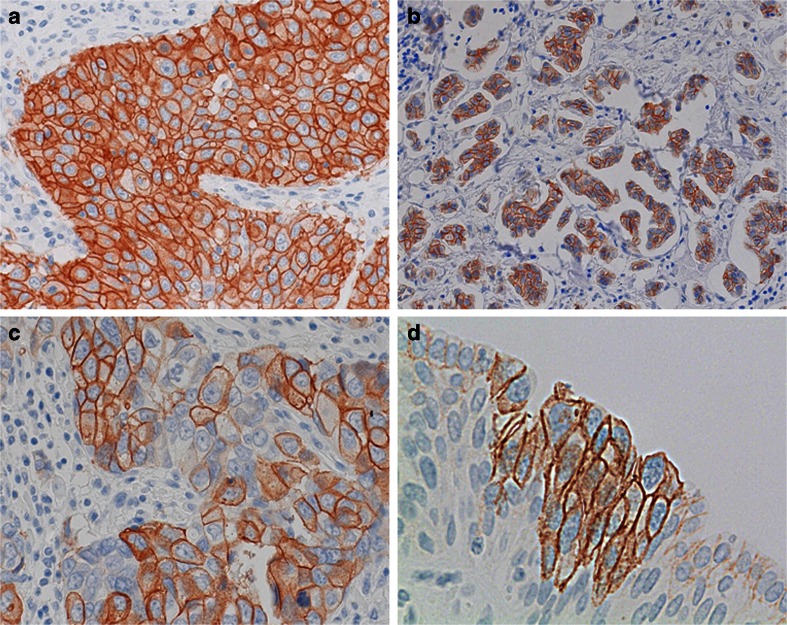
HER2 immunohistochemistry: **a** strong membranous positivity in 100 % of the tumor cells of a conventional urothelial bladder cancer, score 3+; **b** score 3+ in a micropapillary variant; **c** mosaic pattern, score 3+; **d** strong positivity in carcinoma in situ, negativity in the adjacent normal urothelium

Ten out of the thirteen *HER2* amplified tumors (77 %) were scored as strongly HER2 positive (3 +), out of which six tumors (46 %) resulted in 100 %, two tumors (15 %) in 95 %, one tumor (8 %) in 60 %, and one tumor (8 %) in 50 % strong and complete membranous staining (Fig. 4a, b). Interestingly, we infrequently noted a mosaic HER2 expression pattern within overall HER2 expressing tumors scored as 3+ (Fig. 3). Positive cells were found adjacent to negative or incompletely stained cells in alternating patterns (Fig. 4c). No differences in staining intensities were noted between morphological diverse components and also not between superficial and deep parts of these tumors.

There were no HER2 2+ but three (23 %) HER2 negative tumors: one case (8 %) was scored as 1+ with a 50 % weak, incomplete membranous staining and two score 0 cases (15 %) with incomplete weak membranous immunohistochemical reaction in less than 10 % of the tumor cells.

Two cases comprised areas of carcinoma in situ where we noted a strong immunohistochemical reaction throughout the lesion (Fig. 4d).

**Fig. 5:**
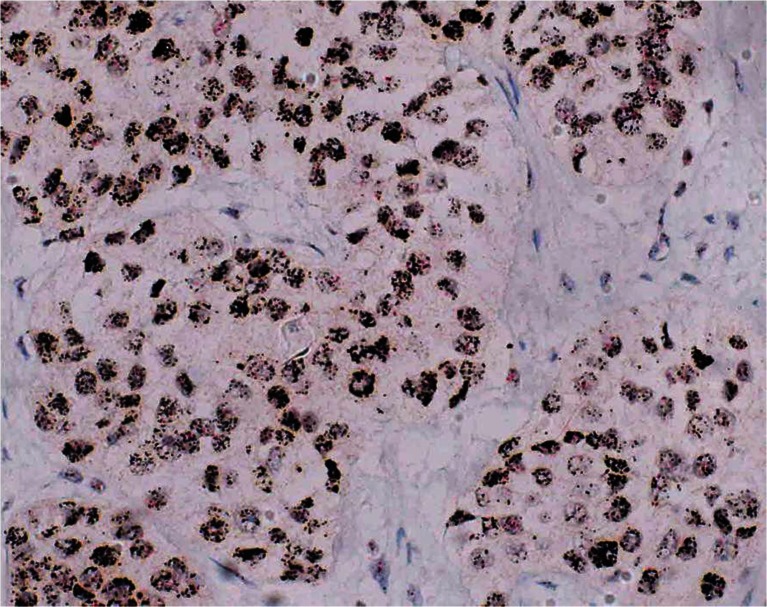
In situ hybridization showing Her2 amplification in 100 % of the neoplastic urothelial bladder cancer cells (*HER2* gene = *black*, centromere 17 = *red*)

We reassessed the relation of HER2 protein overexpression and amplification status on two consecutive large sections. The immunostain showed 100 % strongly positive neoplastic cells (score 3+) and the dual ISH stain presented *HER2* amplification in all cancer cells (Fig. 5).

### HER2 mutation analysis

**Fig. 6:**
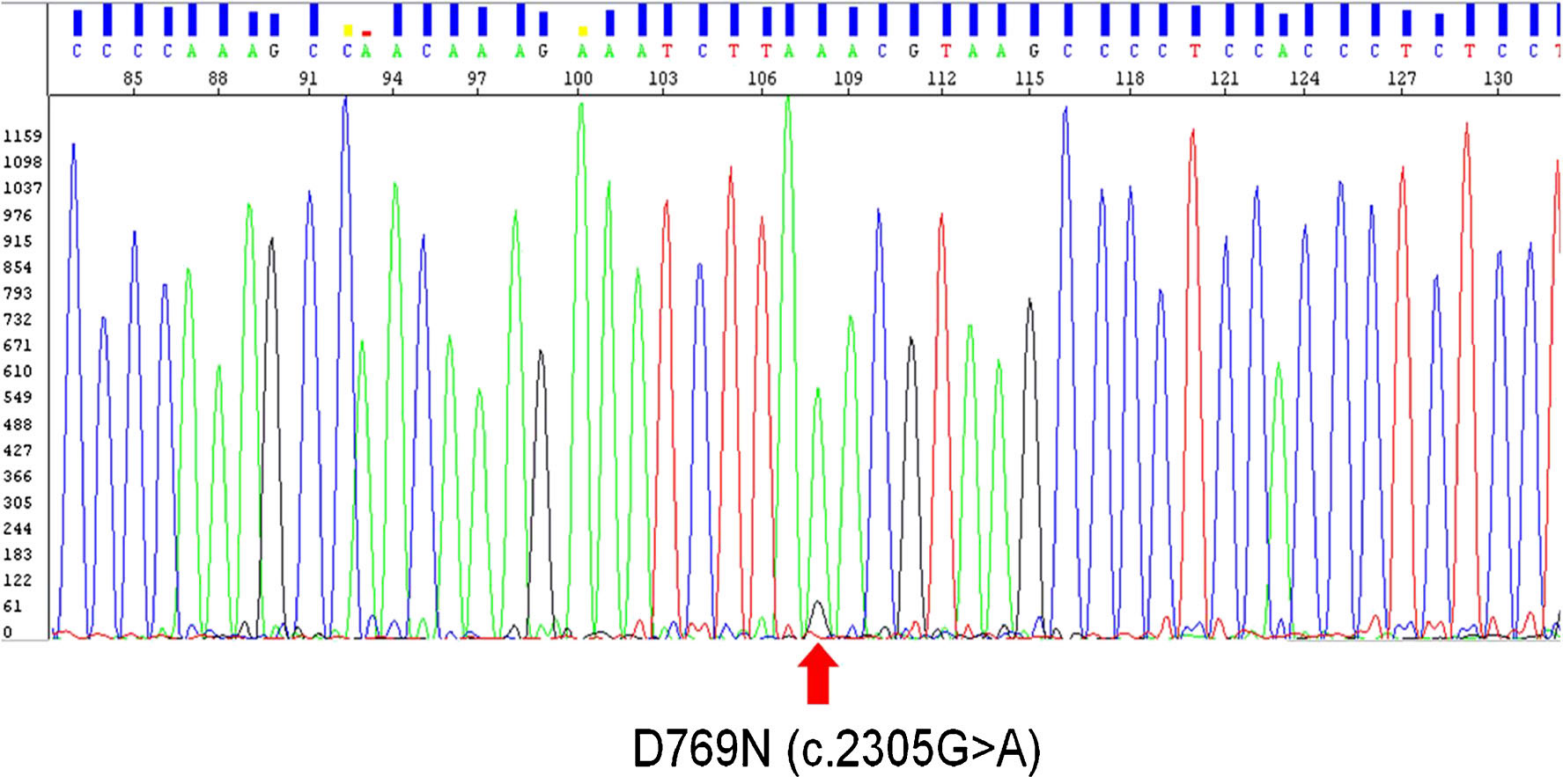
D769N (c.2305G > A) mutation in Exon 19 of the *HER2* gene

The exons 19 and 20 of the *HER2* gene were sequenced in all *HER2* amplified and non-amplified tumors to assess if some of them contain activating mutations within these regions. None of the tumors contained a mutation in exon 20. However, one *HER2* amplified tumor harbored a D769N mutation (c.2305G > A) in exon 19 (Fig. 6). This mutation is located in the αC helix of the catalytic domain of the enzyme.

## Discussion

Specific genetic alterations in cancers may be associated with particular morphological features as shown in colon and prostate cancer [20, 21]. In UBC, there is currently only one comparable investigation. Ching et al. [22] evaluated the particularly aggressive micropapillary variant of urothelial carcinoma for HER2 aberrations [22] and demonstrated HER2 protein overexpression (score 2+ or 3+) in 68 % and *HER2* gene amplification in 42 % of their 20 tumors. However, the micropapillary variant with a prevalence of 0.6–6 % is rare in UBC [17, 22, 23], and in general, a component of a conventional UBC. As yet, the morphological spectrum of UBC with *HER2* amplification, which is an aggressive and from a therapeutic point of view a potentially distinct subgroup, has not been described. Better knowledge about its morphology might help to better identify these tumors. Therefore, we first identified *HER2* amplified UBC in our cohort of high-risk, metastasizing tumors and subsequently described their morphology and HER2 expression patterns.

Micropapillary tumor growth was present in 77 % of our *HER2* amplified tumors; 31 % were purely or predominantly micropapillary UBC, the residual tumors had minor micropapillary fractions. The proportion of micropapillary growth in relation to the entire tumor mass in this group was 33 %. This contrasts significantly with the *HER2* non-amplified UBC group, in which micropapillary growth was present in only one tumor and this component occupied just 3 % of the entire evaluated tumor mass in this group. In addition, the *HER2* amplified tumors presented with a significantly higher morphological heterogeneity than the control group, reflected by a higher number of subtype components per tumor, and showed a significantly higher tumor-associated chronic inflammatory infiltrate. Interestingly, the latter is in line with a recent study showing that HER2 overexpression activates multiple inflammatory pathways, especially NF-κB, which is critical to Interleukin-6 (Il-6) expression [24]. Whether or not a relationship exists with the more recently described association of polyoma virus with micropapillary UBC needs further investigation [25, 26]. Taken together, these histomorphological features of *HER2* amplified UBC—frequent micropapillary architecture, morphological heterogeneity and marked tumor-associated chronic inflammation—allow pathologists to better identify this clinically important, particularly aggressive subset of UBC [12] and herewith to contribute to enhanced survival prediction and preselection for potential anti-HER2 therapies in the future.

Only few studies have investigated *HER2* amplification and overexpression simultaneously in all their bladder cancer patients [27–29]. However, they only categorized the tumors according to DAKO scores and did not report the exact percentage and distribution of positive tumor cells. We noted a strong complete membranous immunoreactivity for HER2 in 77 % of our *HER2* amplified tumors. Importantly, most of these tumors showed this staining in virtually all neoplastic cells; only two cases had a partial tumor staining of 60 and 50 % of the tumor cells. This suggests that HER2 overexpression mostly occurs as an early event in tumorigenesis and only rarely in subsequent tumor development. Further evidence for early HER2 overexpression in tumorigenesis was noted in the two patients with residual urothelial carcinoma in situ which were strongly HER2 positive (score 3+). Interestingly, even though our *HER2* amplified group presented with marked heterogeneous morphology, this observation was not reflected in the HER2 expression pattern. There was no HER2 expression difference between the morphologically diverse components of these tumors; in particular, the micropapillary areas did not show a more pronounced immunoreactivity than the other histological components. Notably, 23 % of our *HER2* amplified tumors were immunohistochemically HER2 negative. Similarly, high rates of “false negative” UBC (20–24 %) have been reported by others [30, 29] and were attributed to putative fixation artifacts. Therefore, we specifically investigated HER2 expression intensity along the gradient of diffusion of formalin from the superficial bladder wall to deeper parts. However, there were no increments in staining intensities to be found.

Finally, we tested all UBC for activating *HER2* mutations which have been reported in a small subset of lung and breast cancer [19, 31, 32]. Interestingly, we detected a D769N mutation in a *HER2* amplified tumor sample. To our knowledge, this mutation has not been described so far, also not in the series of Ross et al who tested 15 micropapillary UBC for mutations [33]. However, two mutations, D769H and D769Y, occurring at the same amino acid position were described in breast cancer [31]. Both mutations conferred constitutive activity of the HER2 kinase. Cell lines bearing these mutations revealed increased HER2, EGFR, and PLCγ phosphorylation and had more rapid tumor growth in xenograft models compared to the wild type control. In addition, both mutations conferred sensitivity to the HER2 inhibitor lapatinib [31]. The authors suggested that activation of HER2 by these mutations may be due to loss of the acidic side chain at D769, or alternatively, due to an aromatic ring introduced by histidine or tyrosine, respectively. Asparagine and tyrosine can often been substituted without affecting protein function since both amino acids contain uncharged polar side chains. If D769N mutation, like D769Y mutation, induces HER2 activity, has to be confirmed experimentally.

In conclusion, the aggressive *HER2* amplified subtype of UBC shows specific histomorphological features—frequent micropapillary architecture, morphological heterogeneity, and marked tumor-associated chronic inflammation—that may allow identifying them with high accuracy. Approximately three-quarters of these tumors overexpress HER2 strongly. This is promising for targeted anti-HER2 therapies.

## References

[1] Schechter AL, Stern DF, Vaidyanathan L, Decker SJ, Drebin JA, Greene MI, Weinberg RA (1984). The neu oncogene: an erb-B-related gene encoding a 185,000-Mr tumour antigen. Nature.

[2] Zwick E, Bange J, Ullrich A (2001). Receptor tyrosine kinase signalling as a target for cancer intervention strategies. Endocr Relat Cancer.

[3] Lenferink AE, Busse D, Flanagan WM, Yakes FM, Arteaga CL (2001). ErbB2/neu kinase modulates cellular p27(Kip1) and cyclin D1 through multiple signaling pathways. Cancer Res.

[4] Yarden Y, Sliwkowski MX (2001). Untangling the ErbB signalling network. Nat Rev Mol Cell Biol.

[5] Wen XF, Yang G, Mao W, Thornton A, Liu J, Bast RC, Le XF (2006). HER2 signaling modulates the equilibrium between pro- and antiangiogenic factors via distinct pathways: implications for HER2-targeted antibody therapy. Oncogene.

[6] Nicholson RI, Gee JM, Harper ME (2001). EGFR and cancer prognosis. Eur J Cancer.

[7] Slamon DJ, Clark GM, Wong SG, Levin WJ, Ullrich A, McGuire WL (1987). Human breast cancer: correlation of relapse and survival with amplification of the HER-2/neu oncogene. Science.

[8] Sauter G, Lee J, Bartlett JM, Slamon DJ, Press MF (2009) Guidelines for human epidermal growth factor receptor 2 testing: biologic and methodologic considerations. J Clin Oncol 27(8):1323-1333. doi:10.1200/JCO.2007.14.8197 10.1200/JCO.2007.14.8197 19204209

[9] Tinoco G, Warsch S, Gluck S, Avancha K, Montero AJ (2013). Treating breast cancer in the 21st century: emerging biological therapies. J Cancer.

[10] Smyth EC, Cunningham D (2012). Targeted therapy for gastric cancer. Curr Treat Options Oncol.

[11] Caner V, Turk NS, Duzcan F, Tufan NL, Kelten EC, Zencir S, Dodurga Y, Bagci H, Duzcan SE (2008). No strong association between HER-2/neu protein overexpression and gene amplification in high-grade invasive urothelial carcinomas. Pathol Oncol Res.

[12] Fleischmann A, Rotzer D, Seiler R, Studer UE, Thalmann GN (2011). Her2 amplification is significantly more frequent in lymph node metastases from urothelial bladder cancer than in the primary tumours. Eur Urol.

[13] Hansel DE, Swain E, Dreicer R, Tubbs RR (2008). HER2 overexpression and amplification in urothelial carcinoma of the bladder is associated with MYC coamplification in a subset of cases. Am J Clin Pathol.

[14] Lae M, Couturier J, Oudard S, Radvanyi F, Beuzeboc P, Vieillefond A (2010) Assessing HER2 gene amplification as a potential target for therapy in invasive urothelial bladder cancer with a standardized methodology: results in 1005 patients. Ann Oncol 21(4):815–819. doi:10.1093/annonc/mdp488 10.1093/annonc/mdp488 PMC2844947 19889613

[15] Wallerand H, Robert G, Bernhard JC, Ravaud A, Ferriere JM (2008). Targeted therapy for locally advanced and/or metastatic bladder cancer. Prog Urol J Assoc Fr Urol Soc Fr Urol.

[16] Wolff AC, Hammond ME, Schwartz JN, Hagerty KL, Allred DC, Cote RJ, Dowsett M, Fitzgibbons PL, Hanna WM, Langer A, McShane LM, Paik S, Pegram MD, Perez EA, Press MF, Rhodes A, Sturgeon C, Taube SE, Tubbs R, Vance GH, van de Vijver M, Wheeler TM, Hayes DF, American Society of Clinical O, College of American P (2007) American Society of Clinical Oncology/College of American Pathologists guideline recommendations for human epidermal growth factor receptor 2 testing in breast cancer. J Clin Oncol 25(1):118–145. doi:10.1200/JCO.2006.09.2775 10.1200/JCO.2006.09.2775 17159189

[17] Amin MB (2009). Histological variants of urothelial carcinoma: diagnostic, therapeutic and prognostic implications. Mod Pathol Off J US Can Acad Pathol.

[18] Buttitta F, Barassi F, Fresu G, Felicioni L, Chella A, Paolizzi D, Lattanzio G, Salvatore S, Camplese PP, Rosini S, Iarussi T, Mucilli F, Sacco R, Mezzetti A, Marchetti A (2006). Mutational analysis of the HER2 gene in lung tumors from Caucasian patients: mutations are mainly present in adenocarcinomas with bronchioloalveolar features. Int J Cancer.

[19] Shigematsu H, Takahashi T, Nomura M, Majmudar K, Suzuki M, Lee H, Wistuba II, Fong KM, Toyooka S, Shimizu N, Fujisawa T, Minna JD, Gazdar AF (2005). Somatic mutations of the HER2 kinase domain in lung adenocarcinomas. Cancer Res.

[20] Jenkins MA, Hayashi S, O’Shea AM, Burgart LJ, Smyrk TC, Shimizu D, Waring PM, Ruszkiewicz AR, Pollett AF, Redston M, Barker MA, Baron JA, Casey GR, Dowty JG, Giles GG, Limburg P, Newcomb P, Young JP, Walsh MD, Thibodeau SN, Lindor NM, Lemarchand L, Gallinger S, Haile RW, Potter JD, Hopper JL, Jass JR, Colon Cancer Family R (2007). Pathology features in Bethesda guidelines predict colorectal cancer microsatellite instability: a population-based study. Gastroenterology.

[21] Mosquera JM, Perner S, Demichelis F, Kim R, Hofer MD, Mertz KD, Paris PL, Simko J, Collins C, Bismar TA, Chinnaiyan AM, Rubin MA (2007). Morphological features of TMPRSS2-ERG gene fusion prostate cancer. J Pathol.

[22] Ching CB, Amin MB, Tubbs RR, Elson P, Platt E, Dreicer R, Fergany A, Hansel DE (2011) HER2 gene amplification occurs frequently in the micropapillary variant of urothelial carcinoma: analysis by dual-color in situ hybridization. Mod Pathol 24(8):1111–1119. doi:10.1038/modpathol.2011.69 10.1038/modpathol.2011.69 21516078

[23] Perez-Montiel D, Hes O, Michal M, Suster S (2006). Micropapillary urothelial carcinoma of the upper urinary tract: clinicopathologic study of five cases. Am J Clin Pathol.

[24] Hartman ZC, Yang XY, Glass O, Lei G, Osada T, Dave SS, Morse MA, Clay TM, Lyerly HK (2011). HER2 overexpression elicits a proinflammatory IL-6 autocrine signaling loop that is critical for tumorigenesis. Cancer Res.

[25] Bialasiewicz S, Cho Y, Rockett R, Preston J, Wood S, Fleming S, Shepherd B, Barraclough K, Sloots TP, Isbel N (2013) Association of micropapillary urothelial carcinoma of the bladder and BK viruria in kidney transplant recipients. Transpl Infect Dis 15(3):283–289. doi:10.1111/tid.12072 10.1111/tid.12072 23551580

[26] Alexiev BA, Randhawa P, Vazquez Martul E, Zeng G, Luo C, Ramos E, Drachenberg CB, Papadimitriou JC (2013). BK virus-associated urinary bladder carcinoma in transplant recipients: report of 2 cases, review of the literature, and proposed pathogenetic model. Hum Pathol.

[27] Coogan CL, Estrada CR, Kapur S, Bloom KJ (2004). HER-2/neu protein overexpression and gene amplification in human transitional cell carcinoma of the bladder. Urology.

[28] Matsubara H, Yamada Y, Naruse K, Nakamura K, Aoki S, Taki T, Tobiume M, Zennami K, Katsuda R, Honda N (2008). Potential for HER-2/neu molecular targeted therapy for invasive bladder carcinoma: comparative study of immunohistochemistry and fluorescent in situ hybridization. Oncol Rep.

[29] Simon R, Atefy R, Wagner U, Forster T, Fijan A, Bruderer J, Wilber K, Mihatsch MJ, Gasser T, Sauter G (2003). HER-2 and TOP2A coamplification in urinary bladder cancer. Int J Cancer.

[30] Latif Z, Watters AD, Dunn I, Grigor K, Underwood MA, Bartlett JM (2004). HER2/neu gene amplification and protein overexpression in G3 pT2 transitional cell carcinoma of the bladder: a role for anti-HER2 therapy?. Eur J Cancer.

[31] Bose R, Kavuri SM, Searleman AC, Shen W, Shen D, Koboldt DC, Monsey J, Goel N, Aronson AB, Li S, Ma CX, Ding L, Mardis ER, Ellis MJ (2013). Activating HER2 mutations in HER2 gene amplification negative breast cancer. Cancer Discov.

[32] Stephens P, Hunter C, Bignell G, Edkins S, Davies H, Teague J, Stevens C, O’Meara S, Smith R, Parker A, Barthorpe A, Blow M, Brackenbury L, Butler A, Clarke O, Cole J, Dicks E, Dike A, Drozd A, Edwards K, Forbes S, Foster R, Gray K, Greenman C, Halliday K, Hills K, Kosmidou V, Lugg R, Menzies A, Perry J, Petty R, Raine K, Ratford L, Shepherd R, Small A, Stephens Y, Tofts C, Varian J, West S, Widaa S, Yates A, Brasseur F, Cooper CS, Flanagan AM, Knowles M, Leung SY, Louis DN, Looijenga LH, Malkowicz B, Pierotti MA, Teh B, Chenevix-Trench G, Weber BL, Yuen ST, Harris G, Goldstraw P, Nicholson AG, Futreal PA, Wooster R, Stratton MR (2004). Lung cancer: intragenic ERBB2 kinase mutations in tumours. Nature.

[33] Ross JS, Wang K, Gay LM, Al-Rohil RN, Nazeer T, Sheehan CE, Jennings TA, Otto GA, Donahue A, He J, Palmer G, Ali S, Nahas M, Young G, Labrecque E, Frampton G, Erlich R, Curran JA, Brennan K, Downing SR, Yelensky R, Lipson D, Hawryluk M, Miller VA, Stephens PJ (2014) A high frequency of activating extracellular domain ERBB2 (HER2) mutation in micropapillary urothelial carcinoma. Clin Cancer Res 20(1):68–75. doi:10.1158/1078-0432.CCR-13-1992 10.1158/1078-0432.CCR-13-1992 24192927

